# Exploring the impact of elevated depressive symptoms on the ability of a tailored asthma intervention to improve medication adherence among urban adolescents with asthma

**DOI:** 10.1186/1710-1492-9-45

**Published:** 2013-11-11

**Authors:** Lokesh Guglani, Suzanne L Havstad, Dennis R Ownby, Jacquelyn Saltzgaber, Dayna A Johnson, Christine C Johnson, Christine LM Joseph

**Affiliations:** 1 Pediatric Pulmonary Division, Department of Pediatrics, Children’s Hospital of Michigan, Wayne State University School of Medicine, 3901 Beaubien St, Detroit, MI 48201, USA; 2 Department of Public Health Sciences, Henry Ford Health System, Detroit, MI, USA; 3 Clinical Allergy and Immunology, Georgia Health Sciences University, Augusta, GA, USA

**Keywords:** Asthma, Depression, Medication adherence, Randomized controlled trial, Self-management, Adolescents, Urban

## Abstract

**Background:**

In patients with asthma, medication adherence is a voluntary behavior that can be affected by numerous factors. Depression is an important co-morbidity in adolescents with asthma that may significantly impact their controller medication adherence and other asthma-related outcomes. The modifying effect of depressive symptoms on an asthma intervention’s ability to improve asthma controller medication adherence among urban adolescents with asthma has not yet been reported.

**Objective:**

To assess self-reported symptoms of depression as an effect modifier of the relationship between randomization group and controller medication adherence at 6-month follow-up.

**Methods:**

These analyses use data from a randomized controlled trial (RCT) conducted in Detroit high schools to evaluate a tailored asthma management program. The intervention included referrals to school or community resources for students reporting symptoms of depression and other issues. “Elevated depressive symptoms” was defined as a positive answer to ≥ 5 of 7 questions from a validated tool included on the baseline questionnaire. Self-reported adherence to controller medication was collected at intervention onset (session 1) and at 6-month follow up. Analyses were restricted to students with report of a controller medication at baseline. Logistic regression was used to assess elevated depressive symptoms as an effect modifier of the relationship between randomization group and 6-month adherence.

**Results:**

Of the 422 students enrolled in the RCT, a controller medication was reported at intervention onset by n = 123 adolescents (29%). Analyzing this group, we observed an interaction between elevated depressive symptoms and adherence (p = 0.073). Stratified analysis showed better adherence in treatment group adolescents meeting criteria for elevated depressive symptoms at baseline as compared to the control group (adjusted Odds Ratio [aOR] = 9.50; p = 0.024). For adolescents without elevated depressive symptoms at baseline, differences in adherence by group assignment did not reach statistical significance (aOR 1.40, p = 0.49).

**Conclusions:**

In this sample of students reporting controller medications at baseline, report of elevated depressive symptoms at baseline and randomization to the intervention group was associated with significantly better adherence at 6-month follow up when compared to that of a control group. Larger studies are needed to evaluate the impact of depression on the relationship between adherence and asthma intervention effectiveness.

## Background

There is a significantly higher prevalence of asthma in urban African American and Latino adolescents and these groups are known to have worse asthma-related outcomes than their White counterparts [1]. Asthma control is impacted by a number of factors, including adherence to prescribed regimens. Adherence, as per its definition, is an “active, voluntary and collaborative involvement of the patient in a mutually acceptable course of behavior to produce a therapeutic result”. Adherence is influenced by a number of internal and external factors including patient beliefs and attitudes, disease and therapy-related factors, health system characteristics, and socioeconomic factors [2]. According to the literature, urban adolescents with asthma in general have poor adherence to asthma controller medications [3]. Studies using electronic monitoring of controller medication adherence in adolescents [4,5] have shown 40-50% adherence, with significantly lower rates of adherence in African American adolescents [6].

Depression is a known co-morbidity of asthma; however, few studies provide estimates of depression as co-morbidity in adolescents with asthma. Existing reports suggest the prevalence of depression among adolescents with asthma ranges from 7.2 to 16.3% [7-9]. Depression may impact quality of life in adolescents with asthma. In a previous analysis of Puff City data, we have shown that depressive symptoms significantly impact emotional quality of life [10]. Depression has been associated with medication adherence in adults and in diseases other than asthma [11-13], but the impact of depression on asthma intervention effectiveness with regard to controller medication adherence has not been explored in urban adolescents.

The present analyses explore the impact of elevated depressive symptoms at baseline on the ability of an asthma intervention to improve adherence to controller medications at 6-month follow-up, among urban teens with asthma. The intervention upon which these analyses are based is Puff City, a computer-tailored, web-based program for urban teens with asthma, originally developed and tested in 2001 [14]. An enhanced version was evaluated in Detroit high schools using a randomized controlled trial conducted from 2007–2011 [15]. The subgroup analyses reported here include urban teenagers that were enrolled in the 2007 – 2011 RCT of Puff City, and reported controller medication(s) at intervention onset.

## Methods

**Figure 1:**
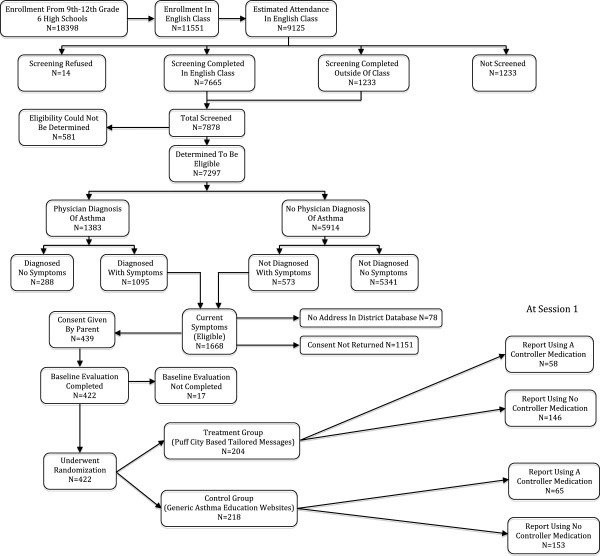
CONSORT Flow diagram for the school-based Puff City randomized controlled trial showing the screening of participants and breakdown of treatment and control groups.

The details of the Puff City randomized controlled trial have been published previously [14-16]. Briefly, to identify students with asthma or asthma symptoms, caregivers of all 9th through 12th grade students of six Detroit public high schools were notified by mail of a Lung Health Questionnaire (LHQ) to be administered during an English class. Parents could opt out of having their child complete the LHQ by signing and returning the letter to the school or by contacting the school. Eligibility to participate in the RCT was based on LHQ responses. To be eligible, the students had to have a physician diagnosis of asthma accompanied by one or more of the following: presence of daytime and/or nighttime symptoms in the last 30 days, medication use for asthma symptoms in the last 30 days, and ≥1 refill(s) of beta-agonists in the last 1 year. Adolescents without a physician diagnosis of asthma were also eligible to participate if they had positive responses to items selected from the International Study of Asthma and Allergies in Childhood (ISAAC) [17], and had symptom frequencies similar to those used in EPR 2 and 3 (e.g., for “mild persistent asthma” criteria from EPR3 include “symptoms ≥ 2 days/week, nighttime awakening ≥ 3-4x/month, and interference with normal activities = minor limitation”) [18,19]. Students identified as eligible for the RCT based on the above criteria were mailed an invitation to participate in the RCT, along with forms for parental written consent and student written assent. Once consent and assent were obtained, a study ID was assigned and entered into the study database. In this way, no student data was shared with investigators until appropriate informed consent was obtained [15]. After a baseline assessment, consenting students were randomized into a treatment or control group (Figure 1). The treatment group underwent a total of 4 computer-based tailored online asthma management sessions that featured topics such as asthma management behaviors (including asthma medication use and adherence, having a rescue inhaler nearby, and smoking cessation and/or reduction), trigger avoidance and basic asthma physiology. The control group was provided access to existing, generic asthma education websites during 4 computer sessions of duration similar to that of the treatment group. Asthma controller medications that were prescribed by a physician were requested from both treatment and control group students at the onset of intervention session 1 using a medication selection module designed for this purpose. The module displayed pictures of medications listed in the Health plan Employer Data and Information Set (HEDIS) measure for asthma called Use of Appropriate Medications for People with Asthma [20], and included corticosteroids, inhaled steroid combinations, leukotriene receptor antagonists, mast cell stabilizers, and antibody inhibitors. Participants were asked to select the asthma medications they were currently taking, if any. All medications reported by the participant were categorized as “controller” or “rescue”.

A referral coordinator was also part of the intervention. The referral coordinator’s task was to assess, refer, and follow-up with students in the intervention group identified to be at-risk of a serious event through a risk assessment report generated by the data management system [16]. Students were contacted if they reported sharing asthma medication, severe asthma symptoms, lack of physician or health insurance, and/or depressive symptoms. Treatment group students with depressive symptoms were usually referred to school-based resources (e.g., school counselor, school social worker, or school-based clinic); and to community-based resources when school resources were not available.

As part of the study protocol, participants received mail and telephone reminders to login to the program and complete a follow-up survey scheduled for 6-months post-baseline. Survey questions collected information on asthma outcomes (e.g., symptom-days, symptom-nights, days of restricted activity), as well as information on controller medication adherence. Follow-up questions were the same for treatment and control group students, although treatment group students could receive additional “booster” messages based on responses to the 6-month survey questions. This study was approved by the Institutional Review Boards of the participating institutions (IRB Protocol #4579) and by the Detroit Public Schools Office of Research, Evaluation and Assessment.

### Study definitions

Depressive symptoms were reported at baseline using 7 questions from the Diagnostic Predictive Scale (DPS) that enquires about symptoms typically associated with depression in the preceding 6-months [21]. The DPS is a result of adaptations to the Diagnostic Interview Schedule for Children (DISC), which is a structured diagnostic instrument specifically designed for use by non-clinicians [22]. The fourth version (DISC-IV) was further adapted to create several shorter scales (including the Diagnostic Predictive Scale used in this study) for use as screening tools for various psychiatric diagnoses, including depression [21]. The DPS has been tested in various populations and has been reported to be an efficient and reliable screening tool [23,24] for children between the ages of 8 to 18 years. Using published cutoff scores established by Lucas et al. [21], elevated depressive symptoms was defined as a positive response to 5 or more questions on the DPS. Adherence to asthma controller medication was defined as self-reported use of the medication on 5 or more days out of last 7 days. Controller medication adherence collected at the 6-month follow-up was the primary outcome of these analyses.

### Statistical analysis

Since the goal of these analyses was to assess the effect of depression on the ability of the intervention to improve controller medication adherence, analyses were restricted to the group of adolescents reporting asthma controller medications at baseline. Logistic regression was used to assess elevated depressive symptoms as an effect modifier of the relationship between randomization group and controller medication adherence at 6-months using the p value of <0.10 as indicating the presence of effect modification and the need to present stratum-specific results [25]. Baseline controller medication adherence was included as a covariate in all logistic regression models. Adjusted odds ratios (aOR) and corresponding 95% confidence intervals were calculated to describe the association between randomization group and controller medication adherence at 6-months.

## Results

The breakdown of the study population is shown in Figure 1. Baseline assessment was completed by 422 adolescents. A total of 58 adolescents in the treatment group (28.4%) and 65 in the control group (29.8%) reported a controller medication at the start of the intervention (Table 1). Among those reporting use of a controller medication at intervention onset, the percentage of adolescents in the treatment and control groups meeting criteria for elevated depressive symptoms was 20.7% (n = 12) and 24.6% (n = 16), respectively, p = 0.60. Controller medication adherence for treatment and control group students at intervention onset was 24.1% (n = 14) and 27.7% (n = 18) respectively.

**Table 1:** Prevalence of elevated depressive symptoms and controller medication adherence at intervention onset (Session 1) for teens in the treatment and control groups

	**Treatment N = 58**		**Control N = 65**		**Odds ratio (95% CI)**	***p *** **value**
**% of teens with elevated depressive symptoms at baseline (n)**	20.7 (12)		24.6 (16)		0.80 (0.34, 1.87)	0.60
**Core behavior and report of medication at session 1**						
Controller medication, adherent ≥ 5 of last 7 days	24.1 (14)		27.7 (18)		0.83 (0.37, 1.87)	0.65
Controller medication, not adherent < 5 of last 7 days	75.9 (44)		72.3 (47)			

At the 6-month follow up, after adjusting for baseline adherence, 22 adolescents in the treatment group (37.9%) reported controller medication adherence, as compared to 17 adolescents in the control group (26.2%) (Table 2). The relationship between elevated depressive symptoms at baseline and controller medication adherence met criterion for the presence of an interaction (p = 0.073) [16,25]. Stratified analysis is presented in Table 3. For adolescents that reported elevated depressive symptoms at baseline, 7/12 (58.3%) in the treatment group reported being adherent to their controller medication at the 6-month follow-up, while 2/16 (12.5%) were adherent in the control group, aOR = 9.5; p = 0.024. For adolescents that did not report depressive symptoms at baseline, medication adherence at 6-month follow-up was only slightly higher among students randomized to the treatment group (32.6%) compared to the controls (30.6%) at the 6-month follow up, aOR = 1.4; p = 0.49.

**Table 2:** Comparison of controller medication adherence at 6 month follow-up for by randomization group for students included in the analysis sample*

	**Treatment N = 58**		**Control N = 65**		Adjusted** odds ratio (95% CI)	***p *** **value**
Core behavior at 6 months**						
Controller medication, adherent ≥ 5 of last 7 days	37.9	(22)	26.2	(17)	2.10 (0.89, 4.92)	0.089
Controller medication, not adherent < 5 of last 7 days	62.1	(36)	73.8	(48)		

*Students enrolled in Puff City and reporting a controller medication at baseline assessment. ** *Adjusted for controller medication adherence at start of intervention.*

**Table 3:** Comparison of controller medication adherence at 6 month follow-up by randomization group and by baseline elevated depressive symptoms, for students included in the analysis sample*

	**Treatment**		**Control**		Adjusted** odds ratio (95% CI)	***p *** **value**
**Meet criteria for elevated depressive symptoms at baseline:**						
Core behavior at 6 months**						
Controller medication, adherent ≥ 5 of last 7 days	58.3	(7)	12.5	(2)	9.50 (1.35, 67.0)	0.024
Controller medication, not adherent < 5 of last 7 days	41.7	(5)	87.5	(14)		
**Do not meet criteria for elevated depressive symptoms at baseline:**						
Core behavior at 6 months*						
Controller medication, adherent ≥ 5 of last 7 days	32.6	(15)	30.6	(15)	1.40 (0.53, 3.67)	0.49
Controller medication, not adherent < 5 of last 7 days	67.4	(31)	69.4	(34)		

*Students enrolled in Puff City and reporting a controller medication at baseline assessment. ** *Adjusted for controller medication adherence at start of intervention.*

## Discussion

The Puff City program uses tailoring to promote positive behaviors such as regular use of controller medications by providing personalized health messages to help address the adolescents’ beliefs, attitudes and barriers to behavior change in addition to referrals from an asthma referral coordinator. Results of these subgroup analyses suggest that the effectiveness of a program to improve adherence to controller medications in urban adolescents with asthma may be significantly impacted by the presence of depressive symptoms. For this reason, it may be worthwhile to address depressive symptoms when treating asthma in order to improve asthma-related outcomes. We did note a modicum of improvement in medication adherence among treatment group students who did not meet criteria for elevated depression at baseline, but a comparison to the control group did not reach statistical significance. Therefore, interventions to improve controller medication adherence in adolescents with and without depressive symptoms may still be needed. Our results may have important implications for designing future interventions specifically targeting improvements in controller medication adherence in urban adolescents with asthma.

Other investigators have also found depression to be a significant determinant of medication adherence in several disorders other than asthma. A recent meta-analysis of 31 studies (18,425 participants) of adults with various chronic conditions reported a 1.76 times greater odds for non-adherence in depressed patients [26]. Another report suggests that approximately 20 to 30 percent of prescriptions are never filled (primary non-adherence) and 50% of medications prescribed for chronic diseases are not taken as prescribed [2]. Medication non-adherence is associated with higher downstream health care costs [27], and can be reduced by improved self-management of chronic disorders such as asthma. We are unaware of any other study that has reported the effectiveness of asthma interventions on controller medication adherence among adolescents with depressive symptoms.

Other co-morbidities have been observed in asthma. Besides depressive symptoms, adolescents with asthma have also been found to have a higher prevalence of anxiety disorder [28] and internalizing behaviors [29]. These are linked through several psychological and biological factors such as the stress of asthma management, medication regimens, and avoidance of allergic triggers; or through cognitive responses to asthma symptoms such as learned helplessness or fear of bodily sensations. In the case of adolescents, having asthma symptoms may induce social anxiety (due to concern for negative evaluation by peers) that can significantly impact asthma-related outcomes [30].

The overall rate of controller medication use in this study was low (29% at baseline) resulting in a small sample available for analyses. There are additional limitations to this study. First, we used self-reported measures of asthma controller medication adherence, which can have questionable validity and reliability [31,32]. We note that self-report of asthma controller medication adherence has been used in national surveys such as National Health and Nutrition Examination Survey (NHANES) [33], and National Asthma Survey (NAS) [34]. Second, we cannot determine which component of the intervention was instrumental in motivating participants to be more adherent, i.e., depressed students received tailored messages about controller medication through the online program in addition to referrals made by the asthma referral coordinator for their depressive symptoms. Moreover, we cannot confirm that students followed up on referrals from the asthma referral coordinator and cannot report on the therapies or advice these students may or may not have received from these referrals. Consequently, we cannot speculate on the *mechanism* by which controller medication adherence was improved among students reporting depressive symptoms at baseline. Third, a sustained intervention effect for controller medication adherence post 6-month follow-up is unknown. Finally, because this study was done in urban adolescents with asthma, the results may only be applicable to other populations with characteristics similar to that of our study population. Given the limitations of this study, additional analyses in larger study samples are needed.

## Conclusions

In these subgroup analyses of data from a RCT to evaluate an online asthma management program for urban adolescents with asthma, students who were randomized to the treatment group and met criteria for elevated depressive symptoms had better controller medication adherence when compared to a control group at 6-month follow up. Adolescents without depressive symptoms at baseline did not show statistically significant improvement in controller medication adherence. Interventions aimed at improving controller medication adherence as part of asthma self-management programs may need to be tailored for adolescents with depressive symptoms.

## Abbreviations

DPS: Diagnostic predictive scales; DISC: Diagnostic Interview Schedule for Children; aOR: adjusted odds ratio; ISAAC: International Study of Asthma and Allergies in Childhood; RCT: Randomized controlled trial; NHANES: National Health And Nutrition Examination Survey; NAS: National Asthma Survey; EPR: Expert panel report; HEDIS: Health plan Employer Data and Information Set; LHQ: Lung health questionnaire.

## Competing interests

There is no personal or financial support or author involvement with organization(s) with financial interest in the subject matter.

## Authors’ contributions

LG participated in discussion of analysis approach, results and interpretation, and prepared the manuscript. SLH conducted statistical analysis, discussed results and interpretation of analysis; reviewed manuscript. CCJ, is a co-investigator with input on study design and implementation, reviewed manuscript. DRO is a co-investigator with input on study design and implementation; reviewed manuscript. CLMJ is the Principal Investigator, participated in discussion of analysis approach and interpretation of results, assisted in preparation of manuscript and manuscript review. All the authors have read and approved the final manuscript.
